# Risk Factors for Back Pain among Southern Brazilian School Children: A 6-Year Prospective Cohort Study

**DOI:** 10.3390/ijerph19148322

**Published:** 2022-07-07

**Authors:** Bruna Nichele da Rosa, Matias Noll, Cláudia Tarragô Candotti, Jefferson Fagundes Loss

**Affiliations:** 1 Escola de Educação Física, Fisioterapia e Dança, Universidade Federal do Rio Grande do Sul, Porto Alegre 90010-150, Brazil; claudia.candotti@ufrgs.br (C.T.C.); jefferson.loss@ufrgs.br (J.F.L.); 2 Instituto Federal Goiano, Goiânia 76300-000, Brazil; matias.noll@ifgoiano.edu.br

**Keywords:** back pain, risk factors, child, adolescent, cohort studies

## Abstract

Risk factors associated with back pain vary in different countries. Given the lack of studies in Latin America, our study aimed to assess back pain and its associated factors for six years in Southern Brazilian school children. All children attending the fifth grade of Teutônia, Brazil, were invited to participate in the study. Only schoolchildren who did not report back pain were included in the first assessment. The schoolchildren completed the Back Pain and Body Posture Evaluation Instrument (BackPEI) during three assessments (2011, 2014, and 2017). BackPEI assesses the presence of back pain and possible associated risk factors (postural, behavioral, and sociodemographic). Generalized estimated equations (GEE) were used to perform a Poisson regression model with robust variance for longitudinal analysis. After six years of follow-up, 75 schoolchildren completed all the assessments. The risk factors associated with back pain were spending more than six hours daily watching television, lifting objects from the ground adopting an inadequate posture, using another backpack type different from those with two straps, and carrying a backpack in an asymmetric way. These results are important in guiding the planning of public policies to minimize this public health problem.

## 1. Introduction

Back pain is a public health issue affecting up to 80% of the adult population and generates both social and economic impacts [1,2]. Back pain is the biggest cost to the health system [2,3]. Moreover, it causes an individual economic impact because it withdraws people from labor prematurely, becoming the fourth cause of disability in 2015 [2,4].

This issue has also been extended to children and adolescents, showing a high prevalence of back pain among this population [5] as well as social and economic impacts [6]. A study in the United States estimated $19.5 billion as an annual cost related to musculoskeletal pain among adolescents aged 10–17 years [7]. This scenario has drawn attention because pain among youth is an important predictor of chronic pain in adulthood [8,9]. Therefore, the early detection of aspects associated with back pain allows the development of preventive and early treatment strategies to avoid persistent pain. Back pain is multifactorial, and seems to be influenced by biological, biomechanical, psychological, emotional, behavioral, and environmental aspects [10,11,12,13]. Therefore, is important to understand as much as possible from all aspects that can influence the occurrence of back pain among children and adolescents to develop a better biopsychosocial approach.

Related specifically to those biomechanical and behavioral aspects, many studies have aimed to assess back pain prevalence and identify the risk factors among children and adolescents [5,6,14,15,16]. However, few longitudinal studies have been conducted in developed countries [5]. 

The prevalence of back pain varies in different countries [5,12,17], and lifestyle habits differ between populations from different cultures. Therefore, the factors associated with back pain can vary between countries [5]. Identifying the lack of evidence regarding this aspect in Latin America [13], our study aimed to assess back pain and its associated factors over six years in Brazilian schoolchildren. This study is a continuation of the longitudinal study by Noll et al. [15], which presented results after three years of follow-up. This study finishes a prospective cohort study, following schoolchildren throughout adolescence.

## 2. Materials and Methods

### 2.1. Sample

This prospective cohort study is a continuation of the Brazilian Longitudinal Study on Back Pain and Posture from Adolescents [13]. All schoolchildren registered in the fifth grade of an elementary school in Teutônia were invited to participate in the screening. Based on the six years of follow-up, only the schoolchildren in the fifth grade who attended high school during the last assessment could be followed throughout the six years. 

According to the 2011 census, when the first assessment was performed [18], Teutônia, a city of Rio Grande do Sul, Brazil, had approximately 32,000 inhabitants, a human development index (HDI) of 0.747 (Brazil’s HDI at the same time was 0.699), 11 primary schools, and 1720 schoolchildren enrolled. Of the 1720 schoolchildren, 401 attended fifth grade. Initially, the assessment team visited all 11 primary schools to obtain authorization to conduct the research. After the school’s authorization, schoolchildren were invited to the classroom, and parents’ or guardians’ authorization were requested. We scheduled a day to assess the schoolchildren whose parents or guardians had signed the written informed consent to participate in the study for the screening. 

**Figure 1:**
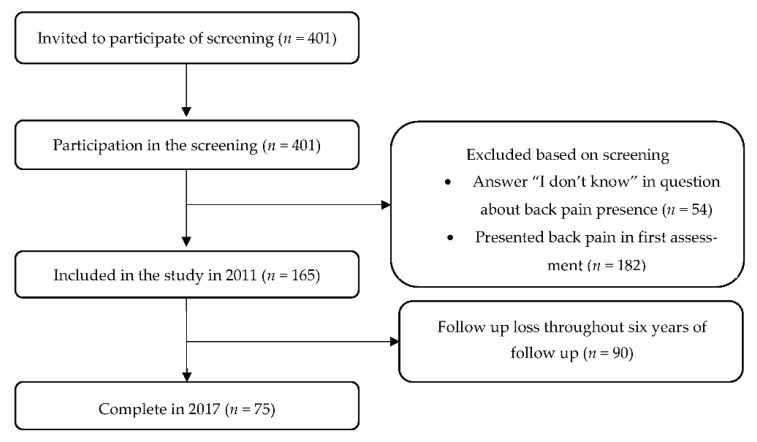
Sample flowchart.

After the screening, we included only those schoolchildren who did not present the outcome (back pain) in the first assessment (2011). Therefore, we excluded schoolchildren who answered “yes” or “I don’t know” to the question of the BackPEI questionnaire “Have you felt (or have been) back pain in the last 3 months?” in the first year of the assessment. Therefore, after the screening, the sample was composed of 165 schoolchildren attending the fifth grade (Figure 1).

This study was performed in accordance with the Helsinki Declaration and was approved by the Ethics Research Committee of the Federal University of Rio Grande do Sul (number 19832). Written informed consent was obtained from all the participants and their parents or guardians.

### 2.2. Data Collection and Analysis Procedures

This prospective cohort study was based on three assessments performed in October 2011, 2014, and 2017. We chose the same month over the three years of assessment because the schoolchildren would always be at the same moment according to the school calendar. Therefore, we sought to avoid different demands and routines caused by different moments in the school calendar (e.g., the exam season).

The answers were obtained from schoolchildren in three years (2011, 2014, and 2017), according to the self-applied BackPEI questionnaire [19]. The BackPEI presents 21 multiple-choice questions and assesses the following aspects: back pain in the last three years (occurrence, frequency, and intensity), hereditary (parents or guardians with back pain), behavioral habits (habit of reading/studying in bed, daily time watching television, using the computer, and sleeping), physical exercise (practice, weekly frequency, and competitive exercise), and postural habits (posture adopted to sleep, to write in the classroom, to use the computer, sit on a bench, lift objects from the ground, and transport school supplies). 

The questions related to postural habits presented figures of schoolchildren performing the tasks, with one female and male version. These figures facilitate identification with the most used posture, leading schoolchildren to choose the answer that best represents him/her. In these questions, among the multiple options to answer, there is the option “Another way/I do not know”. For the analysis, only one option with the figures was considered the correct form to perform daily life activities, while the remaining options were grouped as “incorrect form”. The schoolchildren who answered the option “Another way/I don’t know” were excluded from the analysis of the specific aspect of which they marked this alternative. Back pain intensity was measured using a visual analog scale (VAS) consisting of a 10 cm line. 

On three assessments (2011, 2014, and 2017), the questionnaire was applied in the classroom during the physical education class and was performed by the same assessment team. A group explanation of how to fill in the questionnaire was provided before the assessment.

### 2.3. Statistical Analysis

Statistical analysis was performed using Statistical Package for the Social Sciences (SPSS) software, version 26.0 (IBM, Armonk, NY, USA). Data were analyzed using descriptive and inferential statistics. The chi-square test was used to compare back pain prevalence and back pain frequency between boys and girls on follow-up assessments (2014 and 2017). To compare back pain prevalence and back pain frequency between the assessments (2014 and 2017), the McNemar test was used. The back pain intensity was compared between the assessments (2014 and 2017) using the Student’s *t*-test. 

Generalized estimated equations (GEE) were used to perform a Poisson regression model with robust variance for longitudinal analysis [20,21]. The presence of back pain was the outcome, and the exposure variables included factors related to physical exercise and behavioral, postural, and hereditary habits. Exposure variables with a *p*-value < 0.10 in bivariate analysis were included in the regression model adjusted for sociodemographic variables (sex and age). The risk ratio (RR) and 95% confidence interval (CI) were used. The significance level adopted for all analyses was α = 0.05.

## 3. Results

Of the 401 schoolchildren attending the fifth grade in elementary school invited to participate in the study, 165 were included after the first assessment. After six years of follow-up, 75 schoolchildren completed all assessments (Figure 1), of which 35 (46.7%) were boys, and 40 (53.3%) were girls. The sample characteristics in the first assessment are described in Table 1. The sample that completed the study (*n* = 75) had similar characteristics to those of the sample included in the first assessment (*n* = 165) (Table 1).

The prevalence of back pain was high at the first follow-up (40%). Of the 75 schoolchildren assessed without pain at baseline (2011), 30 schoolchildren presented with back pain in 2014. In the second follow-up (2017), the incidence was 20%. In other words, of the 45 schoolchildren without pain in 2014, nine reported back pain in 2017.

**Figure 2:**
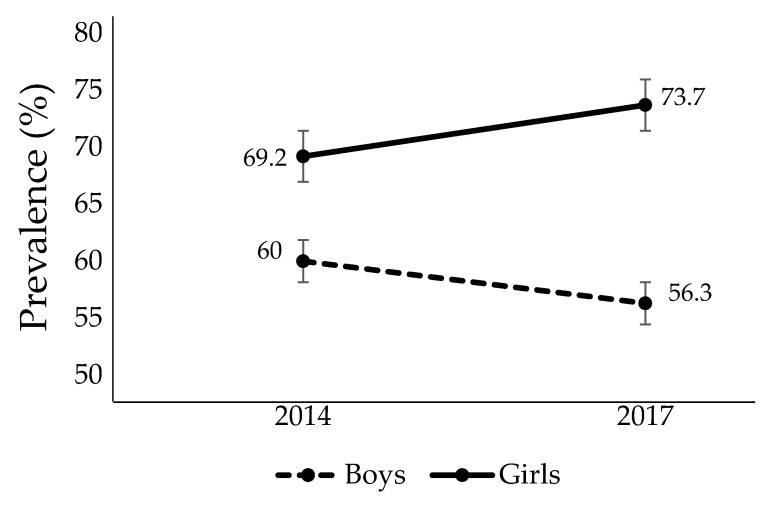
Back pain prevalence among boys and girls in 2014 and 2017.

Figure 2 presents the back pain prevalence in schoolchildren stratified by sex in the follow-up assessments. There were no significant changes in back pain prevalence between the follow-up assessments (2014 and 2017) and the overall sample (*p* = 0.607), boys (*p* = 1.000), or girls (*p* = 0.375) (Figure 2). In both 2014 and 2017, back pain prevalence among boys and girls was not significantly different (2014: *p* = 0.515; 2017: *p* = 0.126).

**Figure 3:**
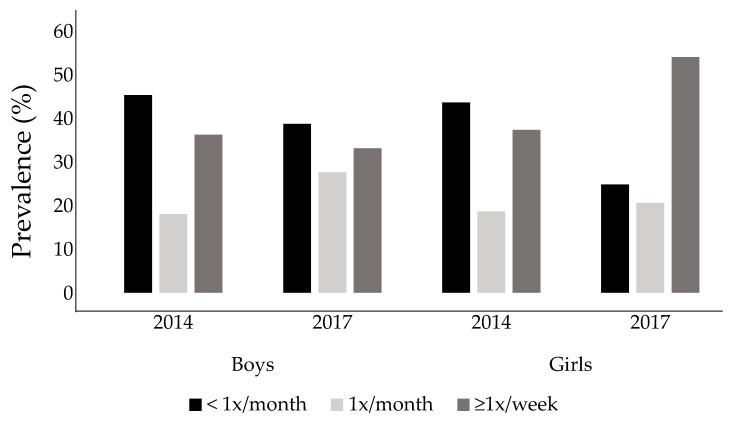
Back pain frequency in two follow-up assessments (2014 and 2017) stratified by sex.

Figure 3 presents the back pain frequency reported by schoolchildren in the follow-up years (2014 and 2017), stratified by sex. There were no significant differences in back pain frequency in the overall sample (*p* = 0.187) or among boys (*p* = 0.392) and girls (*p* = 0.572). Back pain frequency was similar between boys and girls in both 2014 (*p* = 0.996) and 2017 (*p* = 0.399).

Back pain prevented 27.6% of students from performing their daily life activities in the first follow-up year (2014). In 2017, 10.9% of schoolchildren were prevented from carrying out their daily life activities; therefore, there was a significant decrease in this prevalence (*p* = 0.002). In contrast, back pain intensity significantly increased (*p* = 0.002) between the assessments, from 2.5 ± 0.3 to 3.6 ± 0.2. Stratifying the analysis by sex, however, the intensity significantly increased only among girls (girls:2014:2.4 ± 0.3, 2017:3.3 ± 0.2, *p* = 0.012 boys:2014:2.7 ± 0.6, 2017:4.1 ± 0.4, *p* = 0.091). There were no significant differences in back pain intensity between boys and girls in 2014 (*p* = 0.185) or 2017 (*p* = 0.529).

Table 2 presents the data on physical exercise and behavioral, postural, and hereditary aspects. The unadjusted analysis showed that physical exercise and behavioral, postural, and hereditary variables are possible risk factors for back pain (Table 3). After the analysis was adjusted by sex and age, we identified the following risk factors: spending more than six hours daily watching television, lifting objects from the ground adopting an inadequate posture, using another backpack type different from those with two straps, and carrying the backpack in an asymmetric way (Table 3). 

## 4. Discussion

To our knowledge, this is the first prospective cohort study performed in Latin America to assess the presence of back pain and its associated factors among adolescents. Noll et al. [13] presented part of the collected information in a longitudinal study, showing the results from the first two assessments (2011 and 2014) with all schoolchildren assessed. However, this study presents a cohort design, and only those schoolchildren without back pain in the first assessment were included and followed up throughout adolescence, with the aim of identifying the back pain course and its associated factors.

Although no significant differences were found between back pain prevalence in the two follow-up years (2014 and 2017), the back pain incidence was high, with a rate of new cases of back pain of 40% in 2014 and 20% in 2017. The back pain prevalence among boys and girls was high, with more than half of boys and more than 70% of girls presenting back pain prevalence in the last assessment (Figure 2). The frequency of back pain did not show a significant difference between the follow-up years (Figure 3). However, back pain seemed to increase with age, especially among girls, increasing from 2.4 in 2014 to 3.3 in 2017. These results are similar to those of studies performed in other countries, showing that pain tends to increase with increasing age [5,6,8] and affects more girls than boys [8,22]. Another piece of information that calls attention is the high prevalence of back pain found in the screening phase (Figure 1). 45.4% (*n* = 182) of schoolchildren were excluded from the study in the screening phase because they reported back pain in the last three months. This is a high prevalence, but a common scenario, since the recent studies showed a prevalence of around 50% among schoolchildren [12,23,24].

Concerning the risk factors associated with back pain, in the analysis adjusted by sex and age, postural and behavioral habits were identified, such as spending more than six hours daily watching television, lifting objects from the ground adopting an inadequate posture, using another backpack type different from those with two straps, and carrying the backpack in an asymmetric way (not with two stripes on both shoulders). Sedentary behavior has already been documented as a risk factor for back pain among adults, children, and adolescents [13,25,26]. Sedentary behavior is defined as low-energy expenditure activities performed in resting positions [27], including, among other aspects, a long period in the sitting position, which has also been described as a risk factor for back pain [26,28]. It is believed that sedentary behavior can be associated with the presence of back pain not only because it usually involves the sitting position, but also because it can result in the reduction in muscular power, strength, and the ability of intervertebral discs to maintain hydrated, which can causes lesions [29]. In our study, spending more than six hours daily watching television was associated with the presence of back pain. After six years of follow-up, schoolchildren who reported spending more than six hours daily watching television presented a 50% higher risk of presenting back pain compared to schoolchildren who spent less than six hours daily watching television. It is important to mention that only daily time spent watching television and computer use were assessed in our study. However, it is known that adolescents have other sedentary activities in their routine, such as daily time in the classroom and using smartphones or other electronic devices, such as videogames and tablets, and these activities have already been described as possible risk factors for the presence of back pain presence [30]. Therefore, greater attention should be paid to the daily time spent performing all sedentary activities among the school population. Is important to emphasize that schoolchildren from all 11 schools of the municipality participated in the study. Therefore, both children from public and private schools were assessed. Wherefore, is not possible to presume the social context that results in the habit of spending six or more daily hours watching television, since we did not include an assessment of environmental or social aspects. The cause can be many, such as: the lack of parental knowledge about the risks of excessive screen time in many aspects of the children’s life [31], lack of opportunity to participate in extracurricular activities resulting in a long time at home, and so on. However, this draws attention to the long period that the schoolchildren reported spending watching television daily. Therefore, we suggest further investigation based on the environmental and social aspects of schoolchildren.

Another risk factor associated with back pain was inadequate posture adopted to lift objects from the ground (Table 3). Besides the significant association with back pain, it is important to mention the high prevalence of schoolchildren who were reported performing tasks adopting an inadequate posture (Table 2). This behavior has already been observed in other studies that assessed the habits of Brazilian schoolchildren [18,24]. The activity of daily life (ADL) of lifting objects from the ground is a well-discussed topic in back schools, and it is recommended that this ADL be performed with the trunk erect, bending both knees symmetrically, and maintaining the object between the feet [32]. These recommendations are based on scientific evidence showing that lifting an object from the ground itself causes an increase in disc pressure, and when adopting an inadequate posture, the increase in disc pressure is even greater [33].

Postural habits related to carrying school supplies are also associated with back pain. Both the type of backpack used and the method of carrying the backpack are risk factors associated with the presence of back pain (Table 3). Other studies have reported similar results, finding an association between the type of backpack used and the presence of back pain [13,14,34] and with the intensity of back pain [35]. Our study showed that using another type of backpack different from the backpack with two stripes and carrying the backpack in an asymmetric posture (not using the two stripes on both shoulders) increases the risk of presenting back pain (Table 3). It is important to note that this ADL is performed by children and adolescents at school age five days a week, at least twice a day. Therefore, it is important to minimize the undesirable effects of ADL if it performs improperly. Previous studies have shown that carrying a backpack with a symmetric posture facilitates spinal stability [36], and an asymmetric posture can predispose to postural changes that can hinder the absorption of loads correctly by the spine [37].

### Limitations and Strengths

Pain, defined by the International Association for the Study of Pain (IASP), is “an unpleasant sensory and emotional experience associated with actual or potential tissue damage, or described in terms of such damage” [38]. Therefore, it is multifactorial and influenced not only by biomechanical, but also by psychological, emotional, environmental, and social aspects. Is important to highlight that only by taking into account all the aspects that influence back pain occurrence among children and adolescents can a better approach be developed to treat the persistent pain [39]. Thus, a limitation of our study is that we assessed only a part of daily life habits that can be associated with the presence of back pain among a variety of aspects. We did not assess possible sociocultural and psychological factors, focusing only on the biomechanical aspects of postural factors and daily life habits. In addition, regarding the characteristics of pain, we only asked the children about the presence of “back pain”, without specifying the location of pain, which can be a limitation, because the location of the pain can vary among the school children, as well as during the follow-up period.

Moreover, regarding the postural aspects, we assessed it from a biomechanical perspective, choosing one from the multiple alternatives of answers from the BackPEI questionnaire as the correct posture, and grouping the remaining as “incorrect posture”. The correct posture was chosen based on that biomechanically leads to less stress on the joints [40]. In addition, is important to highlight that the relationship between posture and back pain is controversial [41], and is important to pay attention not only to the posture adopted during the ADLs, but also to the period remaining in each posture, an aspect that we did not assess. Still, about the characteristics of the instrument used, the BackPEI is a self-applied questionnaire. Therefore, all the information used in this study was reported by the school children, including their high and weight. This is another limitation, because many school children did not report this information, or reported it wrongly, and the aspects related to body mass index were not assessed as a possible risk factor.

Another limitation was the final sample, composed of 75 schoolchildren who completed the follow-up period. Our aim was to develop an epidemiological study in southern Brazil. However, from all 401 schoolchildren possible to follow throughout the six years, only 165 fulfilled the inclusion criteria. Therefore, our initial sample was already small. Conversely, this cohort study followed the schoolchildren throughout the school stage (pre-adolescence and adolescence) using a validated tool (BackPEI). However, to represent the Brazilian context, more studies developed in other Brazilian regions are needed.

However, the postural habits associated with back pain presented a high prevalence of schoolchildren performing tasks adopting an inadequate posture and other postural habits, such as the posture in the sitting position to write in the classroom, using the computer, and sitting on a bench (Table 2). Therefore, we see the need to educate children and adolescents at the school stage to prevent back pain early and prevent other musculoskeletal complaints. Back schools seem to be a good option since the results have shown the efficacy of educating children about the health of their spine and healthier postural habits [42,43]. 

## 5. Conclusions

Our results showed a high incidence of back pain among Brazilian schoolchildren followed up for six years. Furthermore, the risk factors associated with back pain were spending more than six hours daily watching television, lifting objects from the ground adopting an inadequate posture, using another backpack type different from those with two straps, and carrying the backpack in an asymmetric way (not with two stripes on both shoulders).

## Figures and Tables

**Table 1:** Sample characterization (baseline).

	Included (*n* = 165)		Complete (*n* = 75)	
	Boys (*n* = 96)	Girls (*n* = 69)	Boys (*n* = 35)	Girls (*n* = 40)
Age (Years)	12 ± 0.2	11.6 ± 0.1	11.6 ± 0.5	11.4 ± 0.5
Body mass (kg)	70.7 ± 14.1	44 ± 1	65.8 ± 8.1	43.3 ± 7.3
Height (cm)	131.9 ± 8.1	132.1 ± 8.9	153.2 ± 8.6	153.3 ± 8.5

**Table 2:** Exposure factors frequency (physical exercise, behavioral, postural, and hereditary habits) and back pain frequency (outcome).

Exposure Factors	Baseline (2011)	Follow Up 1 (2014)		Follow Up 2 (2017)	
	*N* (%)	*N* (%)	Back Pain *n* (%)	*N* (%)	Back Pain *n* (%)
Physical exercise practice					
Yes	63 (84%)	41 (89.1%)	26 (63.4%)	58 (78.4%)	34 (58.6%)
No	12 (16%)	5 (10.9%)	4 (80%)	16 (21.6%)	12 (75%)
Physical exercise frequency ^a^					
1 to 2 days per week	31 (53.5%)	15 (38.4%)	8 (53.3%)	29 (50.9%)	19 (65.5%)
3 to 4 days per week	18 (31%)	18 (46.2%)	12 (66.7%)	18 (31.6%)	8 (44.4%)
5 days or more	9 (15.5%)	6 (15.4%)	4 (66.7%)	10 (17.5%)	5 (50%)
Competitive practice of physical exercise ^a^					
Yes	33 (52.4%)	4 (9.5%)	0 (0%)	9 (15.3%)	5 (55.5%)
No	30 (47.6%)	38 (90.5%)	26 (68.4%)	50 (84.7%)	30 (60%)
Daily time watching television					
0 to 1 h	2 (2.7%)	14 (32.6%)	11 (78.6%)	43 (62.3%)	31 (72.1%)
2 to 5 h	51 (68%)	27 (62.8%)	16 (59.2%)	25 (36.2%)	11 (44%)
6 or more hours	22 (29.3%)	2 (4.6%)	1 (50%)	1 (1.4%)	1 (100%)
Daily time using computer					
0 to 1 h	0 (0%)	17 (43.6%)	12 (70.6%)	35 (59.3%)	23 (65.7%)
2 to 5 h	49 (89.1%)	20 (51.3%)	11 (55%)	20 (33.9%)	13 (65%)
6 or more hours	6 (10.9%)	2 (5.1%)	1 (50%)	4 (6.8%)	1 (25%)
Read or study on bed					
Yes	15 (20%)	13 (28.2%)	6 (46.1%)	26 (44.1%)	19 (73.1%)
Sometimes	33 (44%)	9 (19.6%)	5 (55.5%)	0 (0%)	0 (0%)
No	27 (36%)	24 (52.2%)	18 (75%)	33 (55.9%)	18 (54.5%)
Posture adopted to sleep					
On its side	11 (35.5%)	27 (62.8%)	19 (70.4%)	38 (59.4%)	22 (57.9%)
Face down	17 (54.8%)	11 (25.6%)	7 (43.6%)	23 (35.9%)	17 (73.9%)
Face up	3 (9.7%)	5 (11.6%)	2 (40%)	3 (4.7%)	1 (33.3%)
Daily time sleeping					
8 h or more	17 (29.8%)	16 (39%)	14 (87.5%)	16 (25%)	9 (56.2%)
0 to 7 h	40 (70.2%)	25 (61%)	10 (40%)	48 (75%)	32 (66.7%)
Posture adopted to write in classroom					
Adequate	19 (25.3%)	1 (2.2%)	1 (100%)	7 (9.6%)	3 (42.8%)
Inadequate	56 (74.7%)	44 (97.8%)	28 (63.6%)	66 (90.4%)	42 (63.6%)
Posture adopted to sit on a bench					
Adequate	11 (14.9%)	2 (4.3%)	2 (100%)	4 (5.4%)	2 (50%)
Inadequate	63 (85.1%)	44 (95.7%)	27 (61.4%)	70 (94.6%)	43 (61.4%)
Posture adopted to use the computer					
Adequate	9 (12%)	8 (17.4%)	6 (75%)	8 (11%)	6 (75%)
Inadequate	66 (88%)	38 (82.6%)	23 (60.5%)	65 (89%)	38 (58.5%)
Posture adopted to lift object from the ground					
Adequate	25 (36.2%)	5 (11.6%)	1 (20%)	13 (18.6%)	7 (53.8%)
Inadequate	44 (63.8%)	38 (88.4%)	26 (68.4%)	57 (81.4%)	37 (64.9%)
Backpack used to carry school supplies					
Backpack with two straps	6 (8.3%)	47 (100%)	30 (63.8%)	73 (98.6%)	44 (60.3%)
Another type of backpack	66 (91.7%)	0 (0%)	0 (0%)	1 (1.4%)	1 (100%)
Way to carry the backpack ^a^					
Symmetric	21 (28.8%)	34 (72.3%)	20 (58.8%)	52 (70.3%)	31 (59.6%)
Asymmetric	52 (71.2%)	13 (27.7%)	10 (76.9%)	22 (29.7%)	15 (68.2%)
Parents with back pain					
No	2 (25%)	13 (34.2%)	6 (46.1%)	18 (30%)	8 (44.4%)
Yes	6 (75%)	25 (65.8%)	19 (76%)	42 (70%)	30 (71.4%)

^a^ Related only to the schoolchildren to which the variable was applied.

**Table 3:** Association between back pain (outcome) and exposure factors (physical exercise, hereditary, behavioral, and postural habits).

Exposure Variables	RR Analysis (95% CI)	*p* ^a^	Adjusted RR Analysis (95% CI)	*p* ^a^
Control variables				
Sex				
Male	1	0.097	1	0.158
Female	0.94 (0.88–1.01)		1.01 (0.94–1.09)	
Age (Baseline)				
11	1	**<0.001**	1	0.910
12	1.04 (0.97–1.12)		1.01 (0.8–1.3)	
13	**0.84 (0.8–0.87)**		0.77 (0.64–0.92)	
Factors				
Physical exercise practice				
Yes	1	0.262	-	
No	0.92 (0.81–1.06)			
Physical exercise frequency ^b^				
1 to 2x per week	1	0.994	-	
3 to 4x per week	0.99 (0.9–1.1)			
5x or more	1 (0.87–1.14)			
Competitive physical exercise practice ^b^				
Yes	1	**<0.001**	1	0.274
No	**0.79 (0.73–0.86)**		0.88 (0.69–1.11)	
Daily time watching television				
0 to 1 h	1	**<0.001**	1	**0.007**
2 to 5 h	**1.39 (1.24–1.56)**		1.4 (0.9–1.99)	
6 or more hours	**1.54 (1.38–1.72)**		**1.56 (1.15–2.13)**	
Daily time using computer				
0 to 1 h	1	**<0.001**	1	0.806
2 to 5 h	**1.31 (1.17–1.46)**		1.02 (0.99–1.01)	
6 or more hours	**1.38 (1.17–1.63)**		1.01 (0.99–1.02)	
Read or study on bed				
Yes	1	**0.024**	1	0.345
Sometimes	**1.14 (1.02–1.28)**		1.05 (0.99–1.01)	
No	1.02 (0.91–1.15)		1 (0.98–1.1)	
Posture adopted to sleep				
On its side	1	0.068	1	0.548
Face down	1.07 (0.95–1.21)		1.16 (0.7–1.93)	
Face up	1.21 (1.02–1.42)		1.21 (0.85–1.71)	
Daily time sleeping				
8 h or more	1	0.856	-	
0 to 7 h	0.99 (0.88–1.11)			
Posture adopted to write in classroom				
Adequate	1	0.334	-	
Inadequate	1.1 (0.93–1.22)			
Posture adopted to sit on a bench				
Adequate	1	0.145	-	
Inadequate	1.14 (0.78–1.04)			
Posture adopted to use the computer				
Adequate	1	0,334	-	
Inadequate	1.1 (0.93–1.27)			
Posture adopted to lift object from the ground				
Adequate	1	**<0.001**	1	**<0.001**
Inadequate	**1.82 (1.01–3.27)**		**1.18 (1.1–1.3)**	
Backpack used to carry school supplies				
Backpack with two straps	1	**<0.001**	1	**0.010**
Another type of backpack	**1.43 (1.34–1.54)**		**1.33 (1.1–1.64)**	
Way to carry the backpack ^b^				
Symmetric	1	**0.002**	1	**<0.001**
Asymmetric	**1.14 (1.05–1.23)**		**1.5 (1.2–1.9)**	
Parents with back pain				
No	1	**0.004**	1	0.510
Yes	**0.82 (0.72–0.94)**		0.87 (0.59–1.3)	

Generalized estimated equations were used to perform a Poisson regression model with robust variance. Risk ratio (RR) and 95% confidence interval (95% CI) were used to measure the effects (α = 0.05). The adjusted RR analysis was adjusted for sex and age and included exposure factors (*p* < 0.10, RR analysis). Bold data are statistically significant (*p* < 0.05). ^a^ Statistical significance (*p* < 0.05). ^b^ Related only to schoolchildren to which the variable was applied.

## Data Availability

The data presented in this study are available on request from the corresponding author. The data are not publicly available as we do not have a public repository, but if you are interested, please contact Bruna (bruna.nichele@gmail.com) and we will send you the information.

## References

[1] Hoy D., Bain C., Williams G., March L., Brooks P., Blyth F., Woolf A., Vos T., Buchbinder R. (2012). A systematic review of the global prevalence of low back pain. Arthritis Rheum..

[2] Maher C., Underwood M., Buchbinder R. (2016). Non-specifi c low back pain. Lancet.

[3] Walker B.F., Muller R., Grant W.D. (2003). Low back pain in Australian adults: The economic burden. Asia-Pacific J. Public Health.

[4] Hurwitz E.L., Randhawa K., Yu H., Côté P., Haldeman S. (2018). The Global Spine Care Initiative: A summary of the global burden of low back and neck pain studies. Eur. Spine J..

[5] Calvo-muñoz I., Kovacs F.M., Roqué M., Fernández I.G., Calvo J.S. (2018). Risk Factors for Low Back Pain in Childhood and Adolescence a Systematic Review. Clin. J. Pain.

[6] Kamper S.J., Parma T., Williams C.M. (2017). The prevalence, risk factors, prognosis and treatment for back pain in children and adolescents: An overview of systematic reviews. Best Pract. Res. Clin. Rheumatol..

[7] Groenewald C.B., Essner B.S., Wright D., Fesinmeyer M.D., Palermo T.M. (2014). The economic costs of chronic pain among a cohort of treatment seeking adolescents in the United States. J Pain.

[8] Swain M., Henschke N., Kamper S., Gobina I., Ottová-Jordan V., Maher C. (2014). An international survey of pain in adolescents. BMC Public Health.

[9] Hestbaek L., Leboeuf-Yde C., Kyvik K.O. (2006). Is comorbidity in adolescence a predictor for adult low back pain? A prospective study of a young population. BMC Musculoskelet. Disord..

[10] Miller M.M., Meints S.M., Hirsh A.T. (2018). Catastrophizing, pain, and functional outcomes for children with chronic pain: A meta-analytic review. Pain.

[11] Sá S., Silva A.G. (2017). Repositioning error, pressure pain threshold, catastrophizing and anxiety in adolescents with chronic idiopathic neck pain. Musculoskelet. Sci. Pract..

[12] Miñana-Signes V., Monfort-Pañego M., Bosh-Bivià A.H., Noll M. (2021). Prevalence of low back pain among primary school students from the city of Valencia (Spain). Healthcare.

[13] Noll M., Candotti C.T., Rosa B.N.D., Vieira A., Loss J.F. (2019). Back pain and its risk factors in Brazilian adolescents: A longitudinal study. Br. J. Pain.

[14] Ozdemir S., Gencbas D., Tosun B., Bebis H., Sinan O. (2021). Musculoskeletal Pain, Related Factors, and Posture Profiles Among Adolescents: A Cross-Sectional Study from Turkey. Pain Manag. Nurs..

[15] Sundell C.G. (2019). Low back pain and associated disability in Swedish adolescents. Scand. J. Med. Sci. Sport.

[16] Beales D.J., Smith A.J., Sullivan P.B.O., Straker L.M. (2012). Low Back Pain and Comorbidity Clusters at 17 Years of Age: A Cross-sectional Examination of Health-Related Quality of Life and Specific Low Back Pain Impacts. J. Adolesc. Health.

[17] Kamper S.J., Henschke N., Hestbaek L., Dunn K.M., Williams C.M. (2016). Musculoskeletal pain in children and adolescents. Braz. J. Phys. Ther..

[18] Noll M., Candotti C.T., da Rosa B.N., Loss J.F. (2016). Back pain prevalence and associated factors in children and adolescents: An epidemiological population study. Rev. Saude Publica.

[19] Noll M., Candotti C.T., Vieira A., Loss J.F. (2013). Back Pain and Body Posture Evaluation Instrument (BackPEI): Development, content validation and reproducibility. Int. J. Public Health.

[20] Zou G. (2004). A Modified Poisson Regression Approach to Prospective Studies with Binary Data. Am. J. Epidemiol..

[21] Ballinger G.A. (2004). Using Generalized Estimating Equations for Longitudinal Data Analysis. Organ. Res. Methods.

[22] Joergensen A.C., Hestbaek L., Andersen P.K., Andersen A.-M.N. (2019). Epidemiology of spinal pain in children: A study within the Danish National Birth Cohort. Eur. J. Pediatr..

[23] Fabricant P.D., Heath M.R., Schachne J.M., Doyle S.M., Green D.W., Widmann R.F. (2020). The Epidemiology of Back Pain in American Children and Adolescents. BMC Musculoskelet. Disord..

[24] Noll M., Noll P.R., Neto J.L.R., Leal V.N., da Rosa B.N., Candotti C.T. (2017). Back pain and behavioral habits of high school students: A comparative study of two Brazil’s regions. Rev. Bras. Reumatol. (English Ed.).

[25] Dolphens M., Vansteelandt S., Cagnie B., Vleeming A., Nijs J., Vanderstraeten G., Danneels L. (2016). Multivariable modeling of factors associated with spinal pain in young adolescence. Eur. Spine J..

[26] Mahdavi S.B., Riahi R., Vahdatpour B., Kelishadi R. (2021). Association between sedentary behavior and low back pain; A systematic review and meta-analysis. Heal. Promot. Perspect..

[27] Booth F.W., Lees S.J. (2007). Fundamental questions about genes, inactivity, and chronic diseases. Physiol. Genomics.

[28] Bontrup C., Taylor W.R., Fliesser M., Visscher R., Green T., Wippert P.M., Zemp R. (2019). Low back pain and its relationship with sitting behaviour among sedentary office workers. Appl. Ergon..

[29] Bo Andersen L., Wedderkopp N., Leboeuf-Yde C. (2006). Association between back pain and physical fitness in adolescents. BMC Musculoskelet. Disord..

[30] Bento T.P.F., Cornelio G.P., Perrucini P.d.O., Simeão S.F.A.P., de Conti M.H.S., de Vitta A. (2020). Low back pain in adolescents and association with sociodemographic factors, electronic devices, physical activity and mental health. J. Pediatr. (Rio. J)..

[31] Domingues-Montanari S. (2017). Clinical and psychological effects of excessive screen time on children. J. Paediatr. Child Health.

[32] Noll M., Candotti C.T., Da Rosa B.N., Sedrez J.A., Vieira A., Loss J.F. (2016). Layout for assessing dynamic posture: Development, validation, and reproducibility. Pediatr. Phys. Ther..

[33] Wilke H.J., Neef P., Caimi M., Hoogland T., Claes L.E. (1999). New in vivo measurements of pressures in the intervertebral disc in daily life. BMC Musculoskelet. Disord..

[34] Korovessis P., Koureas G., Zacharatos S., Papazisis Z. (2005). Backpacks, back pain, sagittal spinal curves and trunk alignment in adolescents: A logistic and multinomial logistic analysis. BMC Musculoskelet. Disord..

[35] Noll M., Fraga R.A., da Rosa B.N., Candotti C.T. (2016). Risk factors associated with the intensity of back pain in school children of Teutônia (RS). Rev. Bras. Ciências Esporte.

[36] Amro A. (2009). The effect of school bag weight on pain, posture, and vital capacity of lung of three elementary of elementary school in Bethlehem district on Palestine. Middle East J. Fam. Med..

[37] Goodgold S., Corcoran M., Gamache D., Gillis J., Guerin J., Coyle J.Q. (2002). Backpack Use in Children. Pediatr. Phys. Ther..

[38] IASP Terminology. https://www.iasp-pain.org/resources/terminology.

[39] Lin I., Wiles L., Waller R., Goucke R., Nagree Y., Gibberd M., Straker L., Maher C.G., O’Sullivan P.P.B. (2020). What does best practice care for musculoskeletal pain look like? Eleven consistent recommendations from high-quality clinical practice guidelines: Systematic review. Br. J. Sports Med..

[40] Pivotto L.R., Nichele B., Candotti C.T., Noll M., Vieira A. (2018). Proposition of a General Scoring System to the BackPEI. Head Neck Spine Surg..

[41] O’Sullivan P.B., Caneiro J.P., O’Sullivan K., Lin I., Bunzli S., Wernli K., O’keeffe M. (2020). Back to basics: 10 facts every person should know about back pain. Br. J. Sports Med..

[42] Calvo-Muñoz I., Gómez-Conesa A., Sánchez-Meca J. (2011). Eficacia de los tratamientos de fisioterapia preventivos para el cuidado de la espalda en niños y adolescentes. Revisión sistemática. Fisioterapia.

[43] Calvo-Mũoz I., Gámez-Conesa A., Sánchez-Meca J. (2012). Preventive physiotherapy interventions for back care in children and adolescents: A meta-analysis. BMC Musculoskelet. Disord..

